# Recent Advances in Construction of Polycyclic Natural Product Scaffolds via One-Pot Reactions Involving Alkyne Annulation

**DOI:** 10.3389/fchem.2020.580355

**Published:** 2020-10-15

**Authors:** Liyao Zheng, Ruimao Hua

**Affiliations:** ^1^School of Chemistry and Chemical Engineering, Guangzhou University, Guangzhou, China; ^2^Department of Chemistry, Tsinghua University, Beijing, China

**Keywords:** one-pot reaction, cascade cyclization, alkynes, carbocycles, heterocycles, natural products, step economy, scaffold diversity

## Abstract

Polycyclic scaffolds are omnipresent in natural products and drugs, and the synthetic strategies and methods toward construction of these scaffolds are of particular importance. Compared to simple cyclic ring systems, polycyclic scaffolds have higher structure complexity and diversity, making them suitable for charting broader chemical space, yet bringing challenges for the syntheses. In this review, we surveyed progress in the past decade on synthetic methods for polycyclic natural product scaffolds, in which the key steps are one-pot reactions involving intermolecular or intramolecular alkyne annulation. Synthetic strategies of selected polycyclic carbocycles and heterocycles with at least three fused, bridged, or spiro rings are discussed with emphasis on the synthetic efficiency and product diversity. Recent examples containing newly developed synthetic concepts or toolkits such as collective and divergent total synthesis, gold catalysis, C–H functionalization, and dearomative cyclization are highlighted. Finally, several “privileged synthetic strategies” for “privileged polycyclic scaffolds” are summarized, with discussion of remained challenges and future perspectives.

## Introduction

Natural products and their synthetic analogs have a broad range of biological activities, and they are essential sources of drug and drug candidates (Rodrigues et al., 2016; Huffman and Shenvi, 2019; Newman and Cragg, 2020). They are also a powerful toolkit for studying complex biological systems, including probing protein functions (Milroy et al., 2014; Rizzo and Waldmann, 2014; Gerry and Schreiber, 2018). Among them, polycyclic natural products (PNPs) are particularly attractive for their complex scaffolds with well-arranged functional group, as well as unique responses and diverse interactions with biological systems, which can be treated as a large class of privileged structures for drug discovery (Kim et al., 2014; Garcia-Castro et al., 2016). Complex PNPs such as strychnine continuously spur the development of new synthetic strategies, reactions, and reagents for ring construction (Gaich and Baran, 2010; Cannon and Overman, 2012; Armaly et al., 2015; Jürjens et al., 2015). Successful examples of PNPs in pharmaceutical industry, such as morphine, cortisone, camptothecin, vincristine, and paclitaxel (Taxol), have encouraged the syntheses of natural products derivatives and analogs for investigating quantitative structure–activity relationship.

With increasing demands for high-throughput screening and navigating medicinal relevantly chemical space, development of efficient annulative reactions is a core task aiming for rapid access to molecular complexity, scaffold diversity, and desired function that is comparable or even superior to PNPs (Wender et al., 2008; Lachance et al., 2012; Bostrom et al., 2018). By integrating multistep synthetic sequences in one pot, tedious workup and purification procedures can be avoided, thus reducing the amounts of solvent and waste, as well as saving time, labor, and cost. As a result, both efficiency and sustainability are improved along with the increase of pot economy (Clarke et al., 2007; Hayashi, 2016). Johnson et al. (1971) reported an elegant synthesis of progesterone, in which a biomimetic cation-π cyclizations of an alkyne–polyolefin substrate was achieved for one-pot construction of the entire polycyclic steroid scaffold. This landmark work stimulated the successors to develop a range of cascade/domino/tandem reactions for construction of PNPs (Nicolaou et al., 2003, 2006; Ardkhean et al., 2016; Ciulla et al., 2019). Besides using multifunctional substrates, one-pot cyclizations can be also realized by multicomponent reactions, which are also powerful for synthesis of PNPs and construction of natural product–like molecules in a modular and combinatorial fashion (Touré and Hall, 2009).

Alkyne-based building blocks are widely used for cyclization because of their rich reaction activities and tunable cyclization modes under different conditions (Gilmore and Alabugin, 2011; Alabugin and Gold, 2013; Zeng, 2013; Fensterbank and Malacria, 2014; Boyarskiy et al., 2016; Chen L. et al., 2018). The fruitful alkyne chemistry has been exploited for construction of molecules with various molecular shapes and functional groups, resulting in mighty ability for generating molecular complexity and diversity with high atom economy. Especially, when one of the two π bonds are *syn*-difunctionalized, alkynes can be transformed to endocyclic double bonds and serve as C2 synthons for substituted (aromatic) carbocycles and heterocycles. When both of the π bonds are reacted, alkynes can be employed for construction of sp^3^ centers, which exist in numerous PNPs with three-dimensional scaffolds. Although significant progress has been made for assembly of monocyclic and bicyclic molecules via alkyne annulation (Gulevich et al., 2013), it is still challenging for efficient construction of polyheterocycles. Multiple reactive sites or components may interact on each other, making it difficult to balance the reactivity and selectivity for several successive steps in one pot. In the past two decades, we have witnessed blossoming of new synthetic toolkits including C–H functionalization (Chen and Youn, 2012; Yamaguchi et al., 2012; Gulías and Mascareñas, 2016; Abrams et al., 2018; Sambiagio et al., 2018; Zheng and Hua, 2018; Ghosh et al., 2020), palladium catalysis (Chinchilla and Najera, 2014; Düfert and Werz, 2016; Trost and Min, 2020), and gold catalysis (Fürstner, 2009; Zhang et al., 2014; Dorel and Echavarren, 2015; Pflästerer and Hashmi, 2016; Marín-Luna et al., 2019). These emerging synthetic methodologies enable new cascade reaction in mild conditions via substrate activation (for alkynes, their reaction partners, or both of them), thus expanding the utilization of alkynes for syntheses of polyheterocycles with higher efficiency and diversity.

In this review, recent progress in the past decade (2010–2020) in either total synthesis of natural products and synthetic methodologies for construction of natural product–like molecules are surveyed and illustrated by selected examples. Although scaffolds by connection of several simple rings are also prevalent and important, the polycyclic scaffolds discussed here are mainly those with at least three fused, bridged, or spiro rings. For the syntheses, we would like to focus on cascade cyclizations involving three or more reactive sites or components including at least one alkyne, while transformations in one-pot two-stage fashion are also included. As the efficiency for generation of complexity and diversity is the most concerned issue in this review, the one-pot ring-forming step for assembly of the polycyclic ring systems is highlighted, while detailed total synthesis and common substrate scope will not be shown. Synthesis of PNPs using arynes, which has been well-discussed in a recent review (Takikawa et al., 2018), is not covered herein, except for works using alkynes for *in situ* generation of the aryne intermediates.

## Construction of Polycyclic Carbocycles

In 2010, Ma's group (Zhou et al., 2010) and Echavarren's group (Molawi et al., 2010) independently reported asymmetric total synthesis of (–)-englerin A using a similar strategy, in which one-pot construction of an oxa-bridged carbocycles via gold(I)-catalyzed cascade reaction is the key step. Toste's group (Sethofer et al., 2010) also reported gold(I)-catalyzed enantioselective cascade cyclization for construction of diverse fused scaffolds. These works then added fuel to the gold-catalyzed reactions (Gorin and Toste, 2007; Hashmi, 2007; Li et al., 2008) to stimulate rapid development of cascade cyclization via gold-catalyzed alkyne activation in the past decade (Fensterbank and Malacria, 2014; Zhang et al., 2014; Dorel and Echavarren, 2015; Pflästerer and Hashmi, 2016; García-Morales and Echavarren, 2018; Marín-Luna et al., 2019).

**Figure 1:**
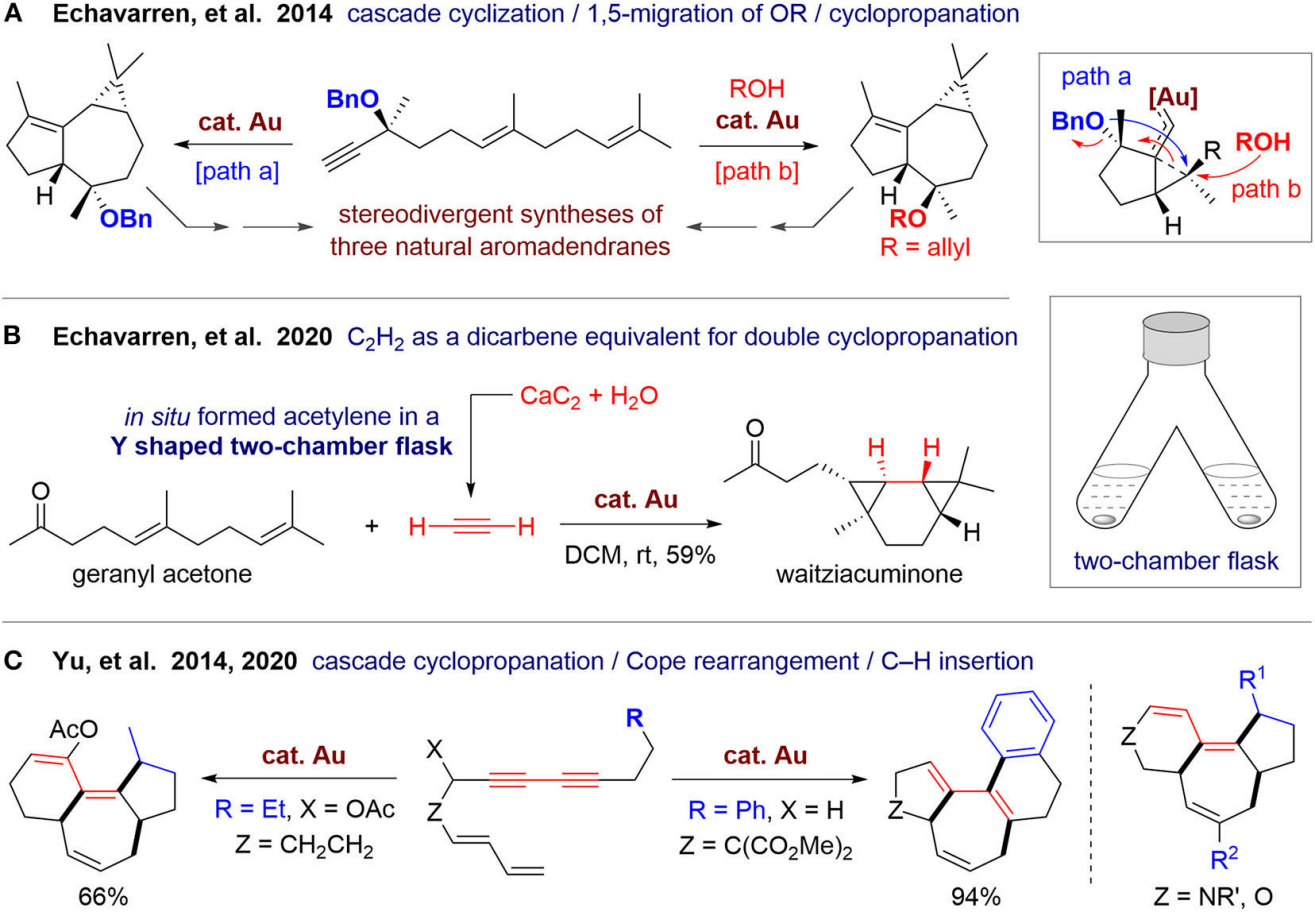
Gold-catalyzed cascade cyclization for syntheses of fused carbocycles.

In 2014, Echavarren's group (Carreras et al., 2014) reported a concise and asymmetric synthesis of three natural sesquiterpenes of the aromadendrane family (Figure 1A). With a linear dienyne as the substrate, a gold(I)-catalyzed cascade cyclization involving 1,5-migration of the OBn group and intramolecular cyclopropanation proceeded efficiently in 5 min at room temperature. The obtained tricyclic product can be further transformed to (–)-epiglobulol and (–)-4β,7α-aromadendranediol in three steps. By adding an external alcohol, product with reverse configuration can be also obtained, and its selectivity can be increased by lowering the temperature. This epimeric product has the stereogenic centers suitable for synthesis of (–)-4α,7α-aromadendranediol. In this protocol, three rings with four new stereogenic centers can be constructed in one pot. This strategy could be extended for the syntheses of both natural products and their non-natural enantiomers to afford a library of polycyclic carbocycles with diverse stereostructures.

Recently, Echavarren's group (Scharnagel et al., 2020) reported a gold(I)-catalyzed double cyclopropanation using acetylene as a unique dicarbene equivalent (Figure 1B). A 3/6/3 fused tricyclic scaffold can be formed in a diastereoselective way by employing 1,5-dienes as substrates, which can be used for one step total synthesis of waitziacuminone from geranyl acetone. In this work, three rings with four new four C–C bonds are constructed in one pot. Notably, the acetylene gas is also produced in one pot, in which the “pot” is an upside-down Y-shaped two-chamber flask. Acetylene is generated *in situ* from calcium carbide and water in one chamber of the flask and then diffuses to the other chamber and dissolved in DCM to participate in the organic reaction. This kind of two-chamber one-flask reaction maintains practical advantages of traditional one-pot reactions and overcomes some disadvantages of reaction control, which would largely increase the modality and extend the boundary of one-pot reactions.

In 2014, Yu's group (Cai et al., 2014) reported a cascade cyclization for polycyclic carbocycles via gold(I)-catalyzed sequential cyclopropanation, cope rearrangement, and C–H functionalization of linear dienediyne substrates. A 6/7/5-fused tricyclic skeleton can be obtained, which exists in some natural diterpenes such as daphnane and tigliane families (Figure 1C). The reaction proceeds well using substrates with nitrogen- or oxygen-containing linkers, but was not suitable for a malonate-tethered substrate. Recently, they (Wang et al., 2020) performed density functional theory calculations and experimental studies to investigate the reaction mechanism, which unveiled the origin of different reactivity of substrates with various tethers. Based on the obtained mechanistic insights, prediction was made and then was tested by experiments, leading to a new methods to access 5/7/5-fused and 5/7/6/6-fused carbocycles via cascade cyclizations involving aliphatic or aromatic C–H insertion, respectively. This work also demonstrates the promising power of computational chemistry for the development of new reactions.

Domino ring-closing metathesis (RCM) can be employed for syntheses of a range of polycyclic carbocycles from well-arranged dienyne substrates. Taxol, one of the most famous anticancer drugs, is among the blockbuster drugs together with its derivatives and to be an attractive synthetic target (Mendoza et al., 2012). Prunet's group (Letort et al., 2016; Ma et al., 2016) investigated the synthesis of taxane and isotaxane derivatives, during which the tricyclic carbocycles were constructed via domino RCM of ene–yne–ene substrates. In the next year, Otero-fraga and Granja (2017) reported one-step assembly of a taxane-like skeleton from an ene–yne–yne–ene substrate. In some cases, the domino RCM sequence may be blocked, and intermediate products were obtained because of high steric hindrance, ring strains, and active functional groups, as well as loss of activity of the catalyst for multiple steps. Therefore, adjustment of the substrates and optimization of the catalytic systems are needed.

**Figure 2:**
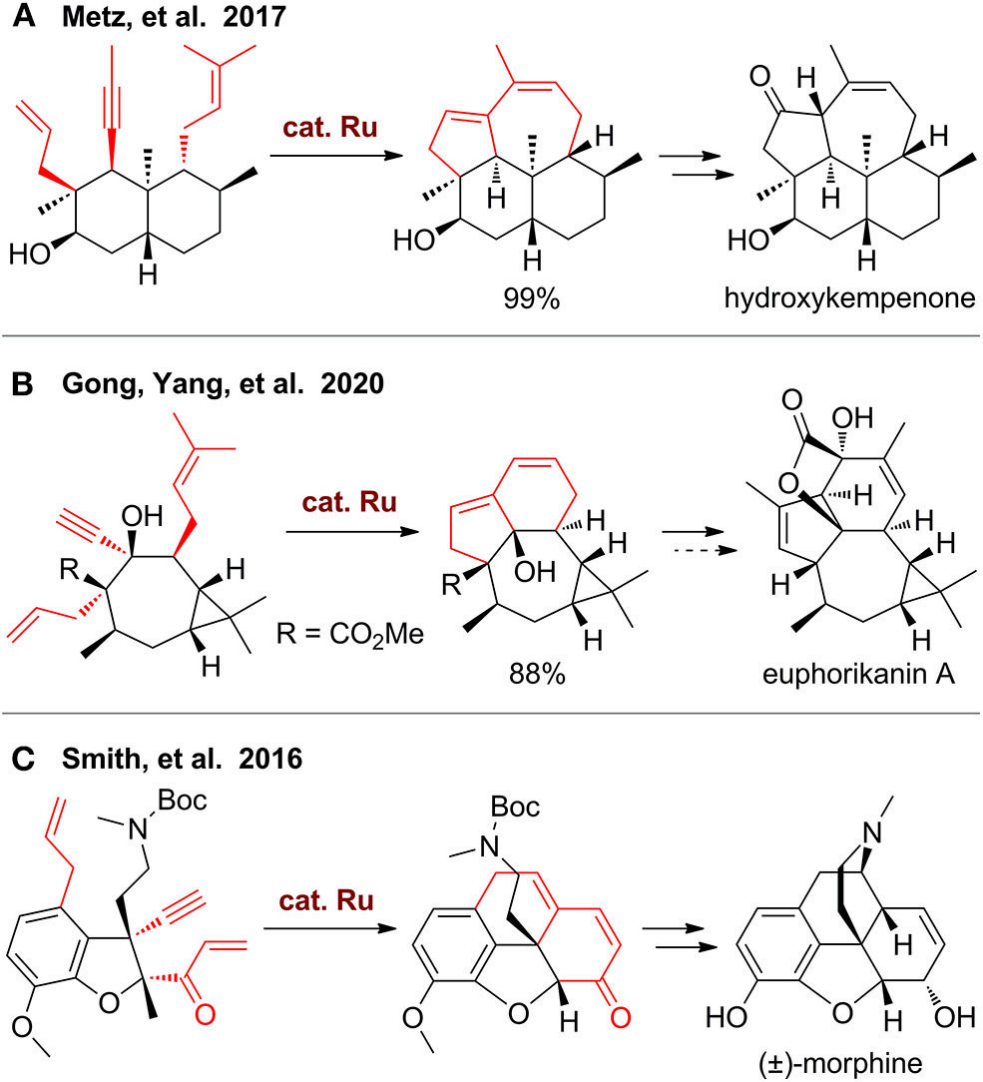
Syntheses of polycyclic carbocycles via domino ring-closing metathesis.

In 2017, Metz's group (Wang Y. et al., 2017) reported the first total synthesis of 3β-hydroxy-7β-kemp-8(9)-en-6-one, a diterpene with 5/6/6/7-fused tetracyclic kempane scaffold (Figure 2A). The key step of this synthesis is a domino RCM from a dienyne-tethered Wieland–Miescher ketone using Grubbs II catalyst, which enables one-pot construction of two fused rings. This single step is remarkably efficient, but synthesis of this substrate needs more than 10 steps. Also, direct transformation of this tetracyclic product to the final target is failed, as the double bond in the seven-membered ring is more reactive, which needs additional steps for protection and deprotection. Very recently, the group of Gong and Yang (Shi et al., 2020) employed a similar strategy for rapid construction of tricyclic scaffold up to gram scale (Figure 2B). The 5/6/7/3-fused tetracyclic ring skeleton of euphorikanin A can be built by further elaboration, but the final total synthesis is impeded during the final lactone formation step.

The domino RCM strategy can also be used for construction of carbocycles fused with heterocycles. In the total synthesis of morphine by Smith's group (Chu et al., 2016), an ene–yne–ene functionalized dihydrobenzofuran was designed, synthesized, and then employed for construction of a tetracyclic scaffold using Hoveyda–Grubbs II catalyst (Figure 2C). This carbocycle further underwent a one-pot C–N bond formation via *in situ* removal of the Boc group with TFA and subsequent neutralization to release a free amino group for conjugated addition. A mixture of two isomers, namely, neopinone, and codeinone were obtained, which can be transformed to codeine and finally to morphine.

**Figure 3:**
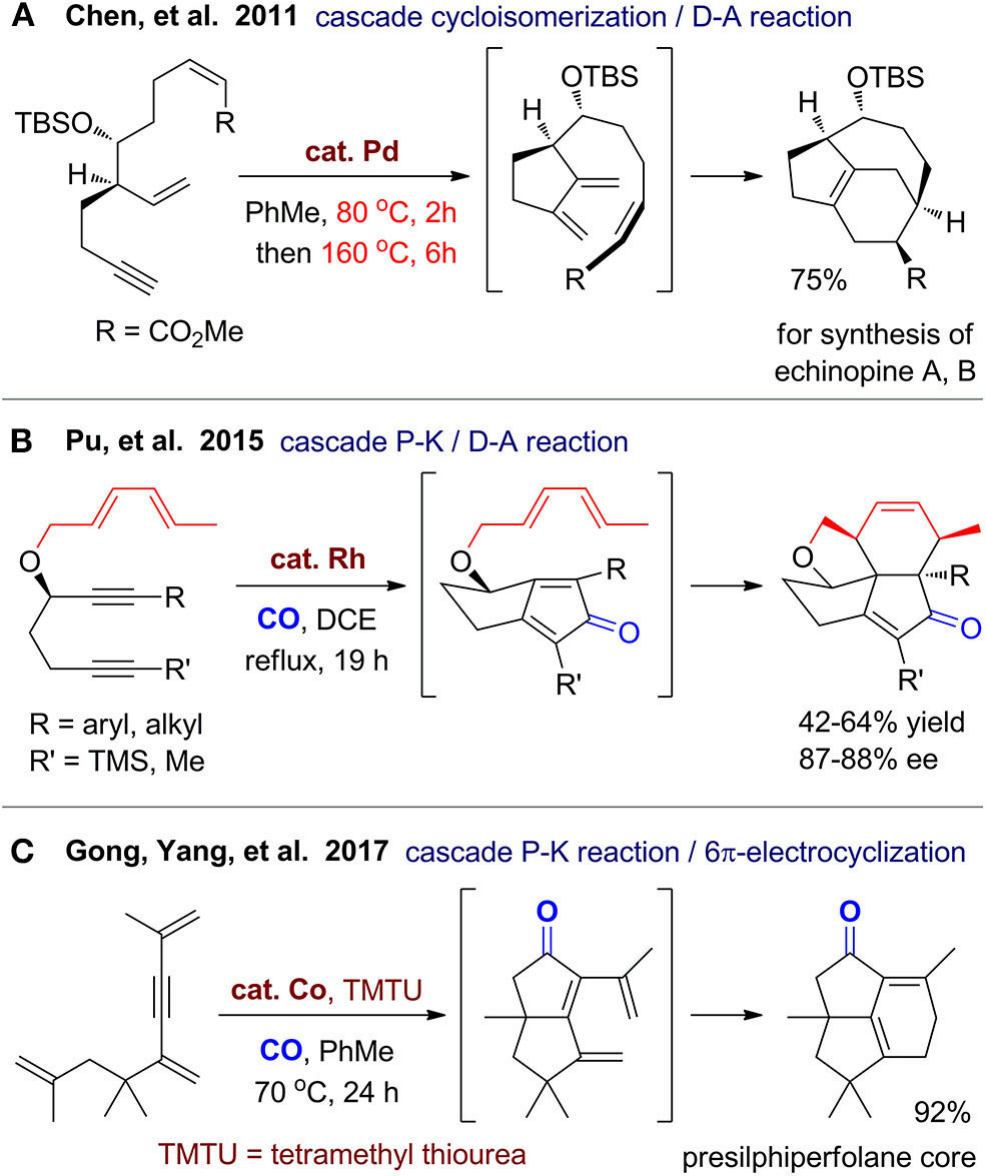
Syntheses of polycyclic carbocycles via cascade cyclization.

By combination of metal-catalyzed annulations with non–metal-catalyzed reactions such as pericyclic reactions, new one-pot synthetic strategies can be realized for construction of diverse polycyclic ring systems. In 2011, Chen's group (Peixoto et al., 2011) reported asymmetric formal syntheses of echinopine A and B with a 5/6/7-fused tricyclic ring system (Figure 3A). A one-pot two-stage reaction was developed to build the tricyclic ring, which involves a palladium-catalyzed enyne cycloisomerization at 80 °C and a subsequent intramolecular Diels–Alder reaction at 160 °C.

As exemplified by Baran's total synthesis of (+)-ingenol (Jorgensen et al., 2013) and Reisman's total synthesis of (+)-ryanodol (Chuang et al., 2016), Pauson–Khand reaction has been proved to be a powerful and practical protocol for construction of polycyclic carbocycles with high efficiency. In 2014, Ying and Pu (2014) reported an efficient construction of pentacyclic ring system of mangicol A from a triene–yne substrate with chiral propargylic ether. Under rhodium(I) catalyst and CO atmosphere, a cascade cycloaddition comprising Pauson–Khand and Diels–Alder reaction proceeds smoothly in a highly chemoselective and stereoselective fashion. The following year, they (Ying et al., 2015) extended this strategy for various branched precursor with two alkynes and a diene group to access several natural product–like tetracyclic carbocycles (Figure 3B). The group of Gong and Yang (Zhang et al., 2017) reported construction of 5/5/6-strained polycyclic system via cobalt/thiourea–catalyzed Pauson–Khand reaction and a cascade 6π electrocyclization (Figure 3C). The obtained tricyclic product can be converted to two presilphiperfolanols and several other tricyclic derivatives. Through ring-opening reactions and functionalizations of this tricyclic product, they (Zhang et al., 2019) recently achieved total syntheses of three sesquiterpenoids, which enriched the application of this reaction. The above works show great potential for expanding the application of Pauson–Khand reaction with other types of reactions especially pericyclic reactions.

## Construction of Polycyclic Heterocycles

### Fused Pyridines and Pyrroles

Nitrogen-containing aromatic rings especially pyridine and pyrrole, as well as quinoline, isoquinoline, and indole are omnipresent in natural products and privileged substructures of a range of PNPs with potent biological activities. In 2014, Wang's group (Yin et al., 2014) reported a total synthesis of ascididemin-type alkaloids, which have a unique pentacyclic scaffold with three pyridine rings. By using propargylamine-derived quinone substrates, a cascade alkyne annulation involving C–H functionalization, deprotection of the Boc group, ketone–amine condensation, and isomerization proceed smoothly under Brønsted acid-promoted and iron(III)-catalyzed oxidative condition. Although two rings are formed in one pot, the synthetic efficiency still has room for improvement. If the preparation of the substrate via oxidative amination can be also integrated to the annulative step, the reaction could be realized in an intramolecular fashion from simple building blocks.

**Figure 4:**
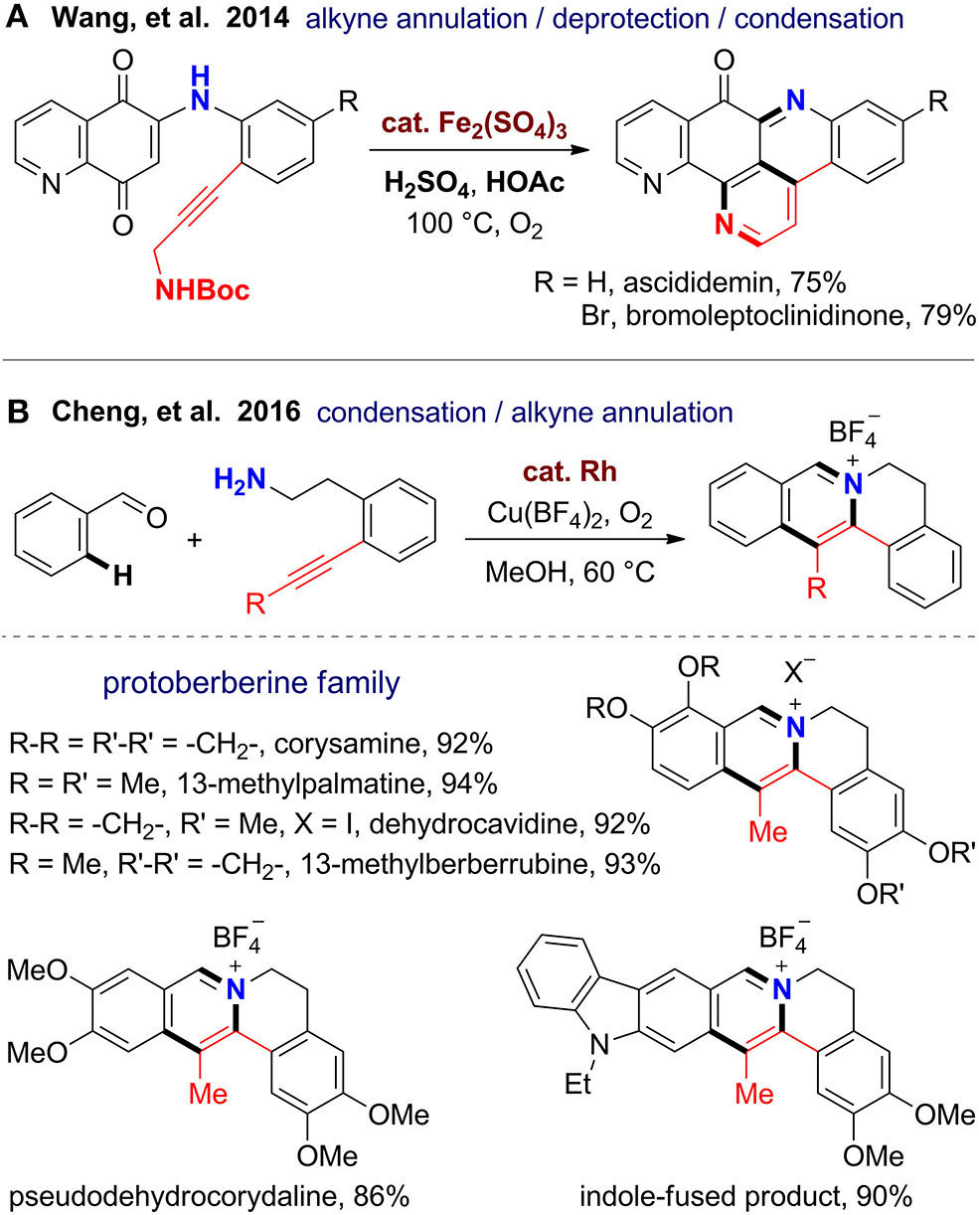
One-pot syntheses of pyridine-fused polycyclic natural products.

Protoberberines are a large class of tetracyclic isoquinoline alkaloids featured with a pyridinium ring. Jayakumar and Cheng (2016) reported a rapid construction of the protoberberine scaffold via one-pot imine formation, C–H activation, and intramolecular alkyne annulation (Figure 4). By using this new method, total syntheses were achieved for several natural protoberberines including corysamine, 13-methylpalmatine, dehydrocavidine, 13-methylberberrubine, and pseudohydrocorydaline from corresponding substituted aryl aldehydes and alkyne–amine bifunctional substrates. This reaction proceeds under mild reaction conditions with O_2_ as the oxidant. The fast formation of imine and intramolecular fashion of the subsequent annulation guaranteed the regioselectivity for alkynes. The reaction has good functional group compatibility and can be scaled up to 1 g. Remarkably, it can be extended to heterocyclic aldehydes and α,β-unsaturated aldehydes to furnish products with indole-fused or truncated protoberberine scaffolds. Also, the obtained protoberberine salts can be transformed to 8-oxyprotoberberines.

**Figure 5:**
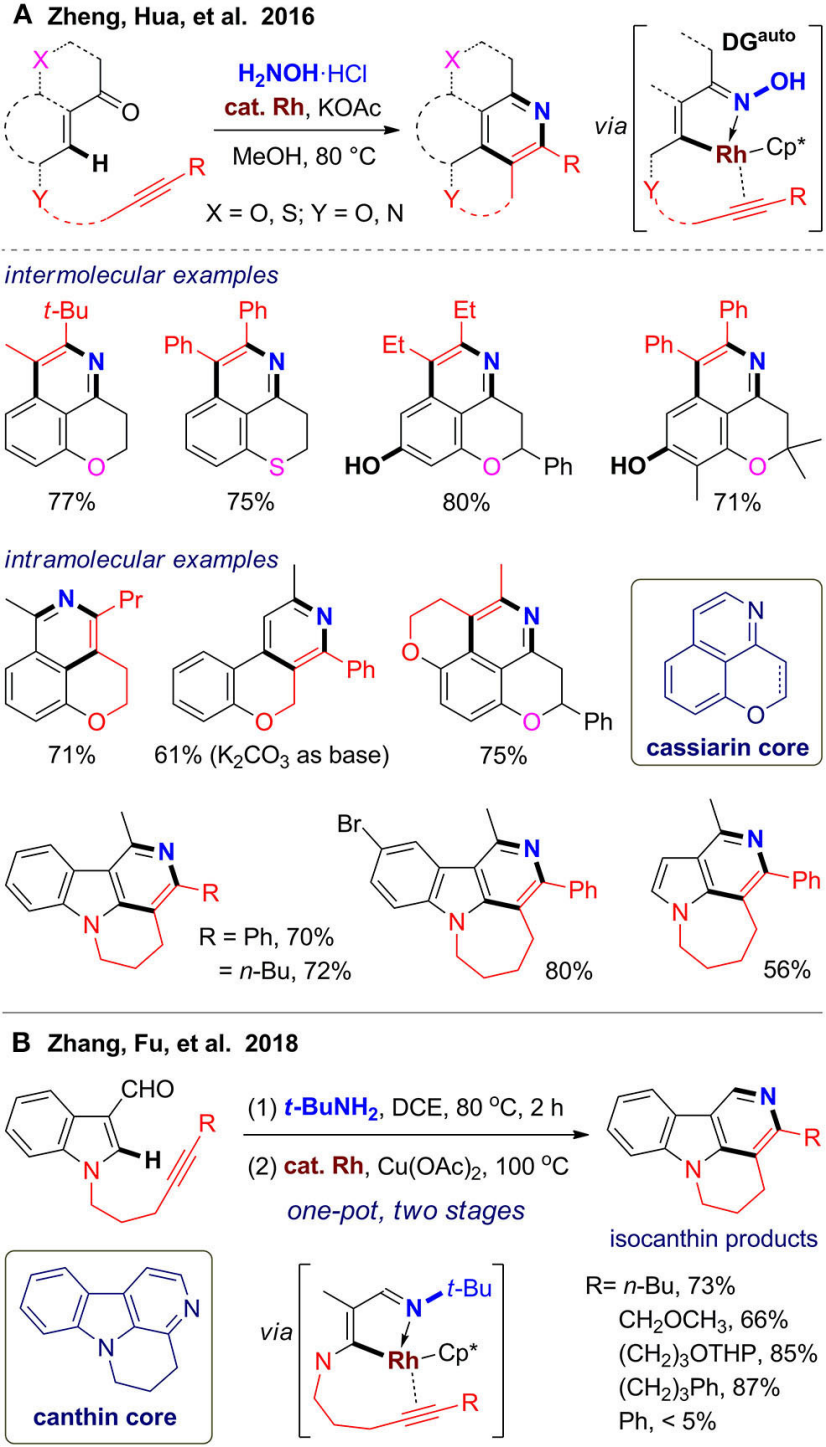
Rhodium-catalyzed one-pot syntheses of heterocycle-fused pyridines via C–H activation.

Inspired by the structure of cassiarins, a class of natural products from *Cassia siamea* with featured tricyclic scaffold, our group (Zheng et al., 2016) devised two strategies for rapid assembly of pyridine-fused heterocycles via intermolecular or intramolecular alkyne annulation via C–H activation assisted by the oxime automatic directing group (Zheng et al., 2012; Zheng and Hua, 2014a) (Figure 5A). With readily available chroman-4-ones as substrates, tricyclic scaffold of cassiarin C can be obtained via *in situ* oxime formation and subsequent C–H activation/annulation, which can be further dehydrogenated to the scaffold of cassiarin A using DDQ. The reaction has excellent regioselectivity for asymmetric alkynes and good functional group compatibility for chroman-4-ones, including tolerance of free hydroxyl group, which also exists in cassiarins. In addition, a thiochroman-4-one is also reacted well to afford sulfur-containing analogs. For the intramolecular protocol, we designed several alkyne–ketone substrates, which can be modular assembled from simple building blocks in one-step. These bifunctional substrates reacted as expected to furnish diverse pyridine-fused polyheterocycles (Figure 5A). Especially, γ-carbolines fused with a six- or seven-membered ring can be synthesized from alkyne-tethered 3-acetyl indoles. By using the above one-pot reactions, a small library of polyheterocycles with cassiarin, isocanthine, or natural product–like scaffold were obtained, which illustrates the power of the C–H activation/annulation for generation structural diversity from simply building blocks.

In 2018, Zhang and co-workers (Chen A. Y. et al., 2018) reported an efficient synthesis of isocanthines using a similar intramolecular strategy (Figure 5B). The reaction proceeds in a one-pot two-stage fashion. Alkyne-tethered indole-3-carboxaldehydes were first transformed to the corresponding *tert*-butylimines at 80 °C for 2 h, followed by addition of catalyst, oxidant, and solvent for further reaction at 100 °C for 16 h. Although excess *t*-BuNH_2_ and stoichiometric copper(II) oxidant were needed, this method enables reaction using aldehyde substrates to give 1-unsubstituted isocanthines, which is limited in our method. These two methods can be also extended to pyrrole derivatives to access fused 5-azaindoles. The above works illustrated the application of C–H activation and intramolecular annulation (Peneau et al., 2018) to construct the pyridine ring with concurrent formation of a fused ring by using *in situ*–formed alkyne-tethered substrates.

**Figure 6:**
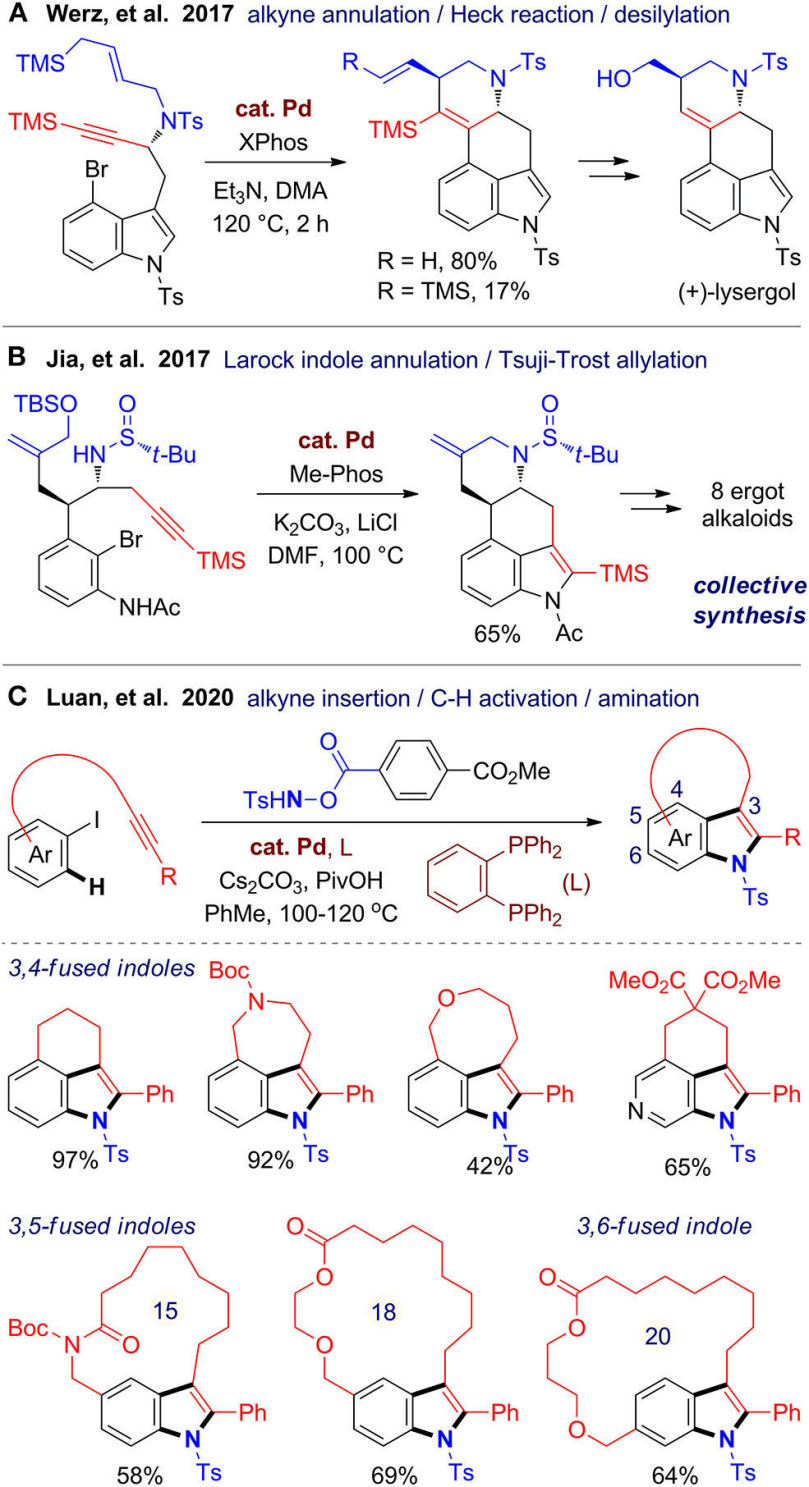
Palladium-catalyzed one-pot synthesis of fused indoles.

Fused indoles are an important class of alkaloids with a range of biological activities (Yuan and Jia, 2018; Connon and Guiry, 2020). In 2017, Werz's group (Milde et al., 2017) reported a stereospecific and enantioselective total synthesis of (+)-lysergol, a representative in the ergot family. The key step is a palladium-catalyzed cascade cyclization relies on the alkyne anti-carbopalladation chemistry developed by the same group (Pawliczek et al., 2015). The cyclization is terminated by a TMS-directed Heck reaction, and the TMS group can also be removed in one-pot in a high ratio, resulting in an exocyclic double bond for further transformation (Figure 6A). In the same year, Jia's group (Liu et al., 2017) accomplished a collective total synthesis of eight ergot alkaloids, in which a new cascade reaction was developed for one-pot assembly of the common B/C/D rings in the tetracyclic ergot scaffold (Figure 6B). This reaction involves an intramolecular Larock indole annulation and a Tsuji–Trost allylation, which are both catalyzed by palladium and can be integrated in one pot.

Very recently, Luan's group (Fan et al., 2020) developed a versatile strategy for one-pot construction of tricyclic fused indoles from alkyne-tethered iodobenzenes and an optimized hydroxylamine as the *N*-source (Figure 6C). This [2 + 2 + 1] annulation is proposed to be proceeded via oxidative addition of aryl iodide to Pd(0) species, followed by intramolecular cyclopalladation involving C–H activation, and an aminative annulation with two C–N bond formation. By variation of the linkers, diverse 3,4-fused indoles with a six-, seven-, or eight-membered fused ring formed smoothly with good functional group tolerance. Moreover, a range of macrocyclic 3,5- and 3,6-fused indoles can be also created, which largely expands the scaffold diversity. Shortly after this work published, two independent works (Cheng et al., 2020; Zhang et al., 2020) were reported using similar catalytic systems for synthesis of 3,4-fused indoles by using *N,N*-di-*tert*-butyldiaziridinone as the *N*-source. By employing this cascade cyclization, Cheng et al. (2020) accomplished a concise synthesis of rucaparib, which is a PARP-1 inhibitor and a Food and Drug Administration–approved cancer medicine.

**Figure 7:**
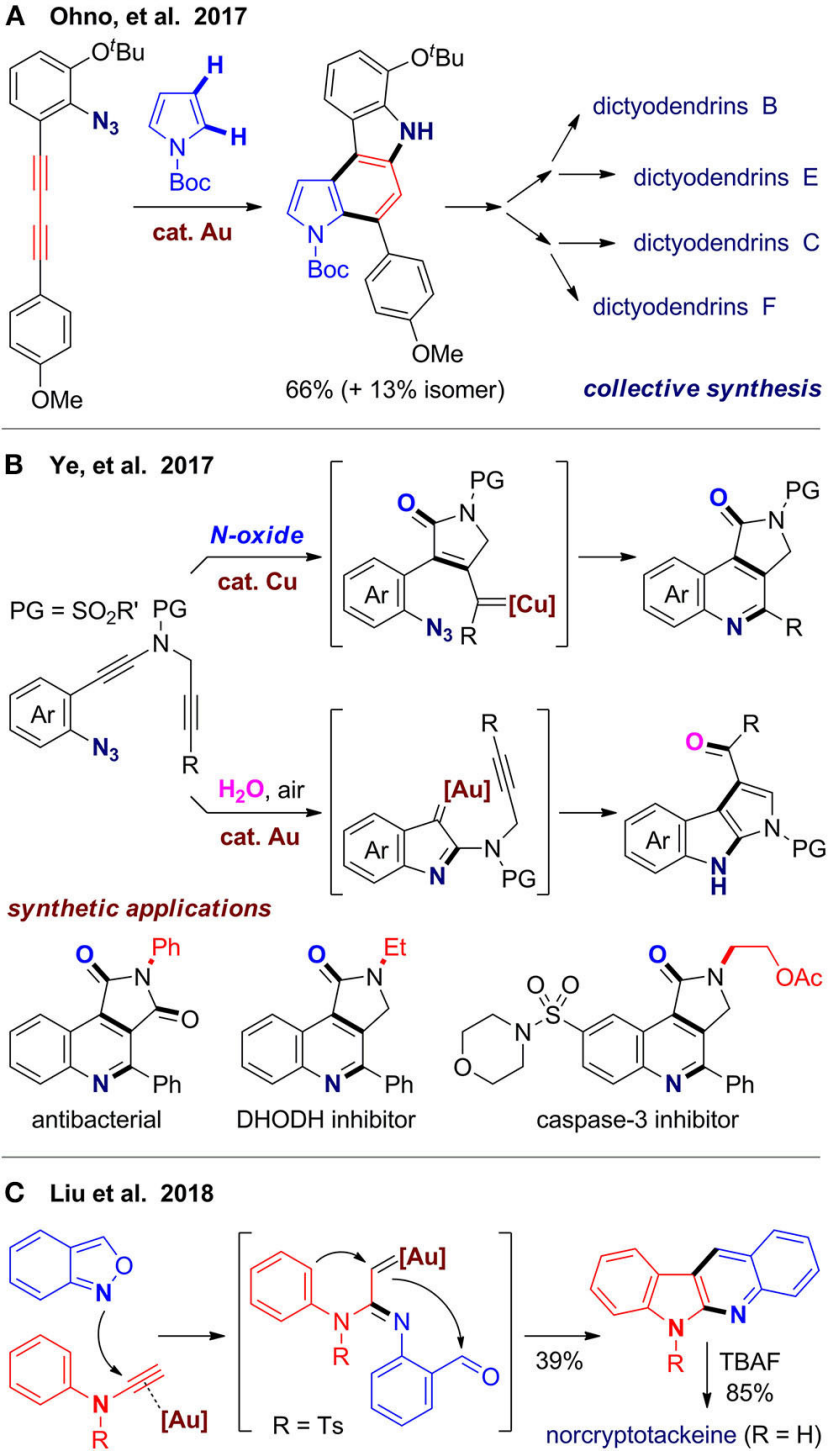
Gold- or copper-catalyzed one-pot synthesis of nitrogen-containing polyheterocycles.

Dictyodendrins are a family polycyclic alkaloids isolated from marine sponges with potent biological activities, which inspired and provided a test platform for new synthetic methods (Zhang and Ready, 2017). Ohno's group (Matsuoka et al., 2017) reported total and formal syntheses of four dictyodendrins, in which the pyrrolo[2,3-*c*]carbazole core is assembled via cascade alkyne annulation. With a cationic gold(I) complex as catalyst, an intramolecular denitrogenative cyclization first occurs from the azide group and its adjacent alkyne, followed by an arylation via intermolecular coupling with pyrrole, and finally intramolecular hydroarylation of the other alkyne to furnish the tetracyclic scaffold (Figure 7A). As C–H bonds at both C2 and C3 sites are reactive in the arylation step, it becomes a key issue to control the regioselectivity. Fortunately, a substrate with alkoxyl substituents reacts well with improved regioselectivity to give a product suitable for divergent syntheses of dictyodendrins B, C, E, and F. The pyrrolo[3,2-*c*]carbazole isomers, which are unwanted byproducts in target-oriented syntheses, would be rather valuable in diverse-oriented syntheses, as they can enrich the skeleton diversity via realignment of privileged substructures of natural products. The same group (Kawada et al., 2018) further extended the strategy to other substrates to synthesize a library of benzo[*c*]carbazoles and indolo[2,3-*c*]carbazoles, which may be useful for development of organic materials.

Ye's group (Shen et al., 2017) designed another type of azido-diyne substrates for divergent syntheses of valuable *N*-heterocycles via controllable cascade cyclization (Figure 7B). With copper(I) as catalyst and N–O bond oxidant, oxidative diyne annulation first occurs, followed by denitrogenative coupling to give pyrrolo[3,4-*c*]quinolin-1-ones. The obtained heterocyclic products can be further transformed to several bioactive molecules, such as antibacterial, DHODH inhibitor and caspase-3 inhibitor. With gold(I) as catalyst and water under air, these substrates undergo a different reaction pathway to afford products with a pyrrolo[2,3-*b*]indole scaffold, which is also a core structure in some bioactive molecules. By using azido-alkyne substrates, Cai et al. (2018) developed a gold(I)-catalyzed bicyclization of alkynes to furnish a tetracyclic framework fused by indole and isochroman, which can be further transformed to diverse fused or spiro polycyclic scaffolds.

In 2018, Liu's group (Tsai et al., 2018) reported a gold(I)-catalyzed cascade reaction of *N*-aryl ynamides with anthranils (benzo[*c*]isoxazoles) for rapid construction of 6*H*-indolo[2,3-*b*]quinoline, in which the isoxazole ring is opened and two fused rings are constructed in one-pot (Figure 7C). This reaction was further used for total synthesis of three natural alkaloids with this scaffold including norcryptotackeine, neocryptolepine, and 11-methylneocryptolepine. By using *N*-benzyl ynamides and anthranils as substrates, Hashmi's group (Zeng et al., 2018) developed a gold(III)-catalyzed synthesis of polyazaheterocycles with a unique dihydroisoquinoline-quinoline fused framework. Interestingly, when *N*-furanylmethyl ynamides are used, functionalized pyrroles, and 1*H*-pyrrolo[2,3-*b*]quinolines can be obtained via a different reaction pathway involving ring-opening reaction of furan. The above works also demonstrated application of ynamides as a bifunctional building block for construction of *N*-heterocycles, which can serve as both a carbon synthon and a nitrogen source.

**Figure 8:**
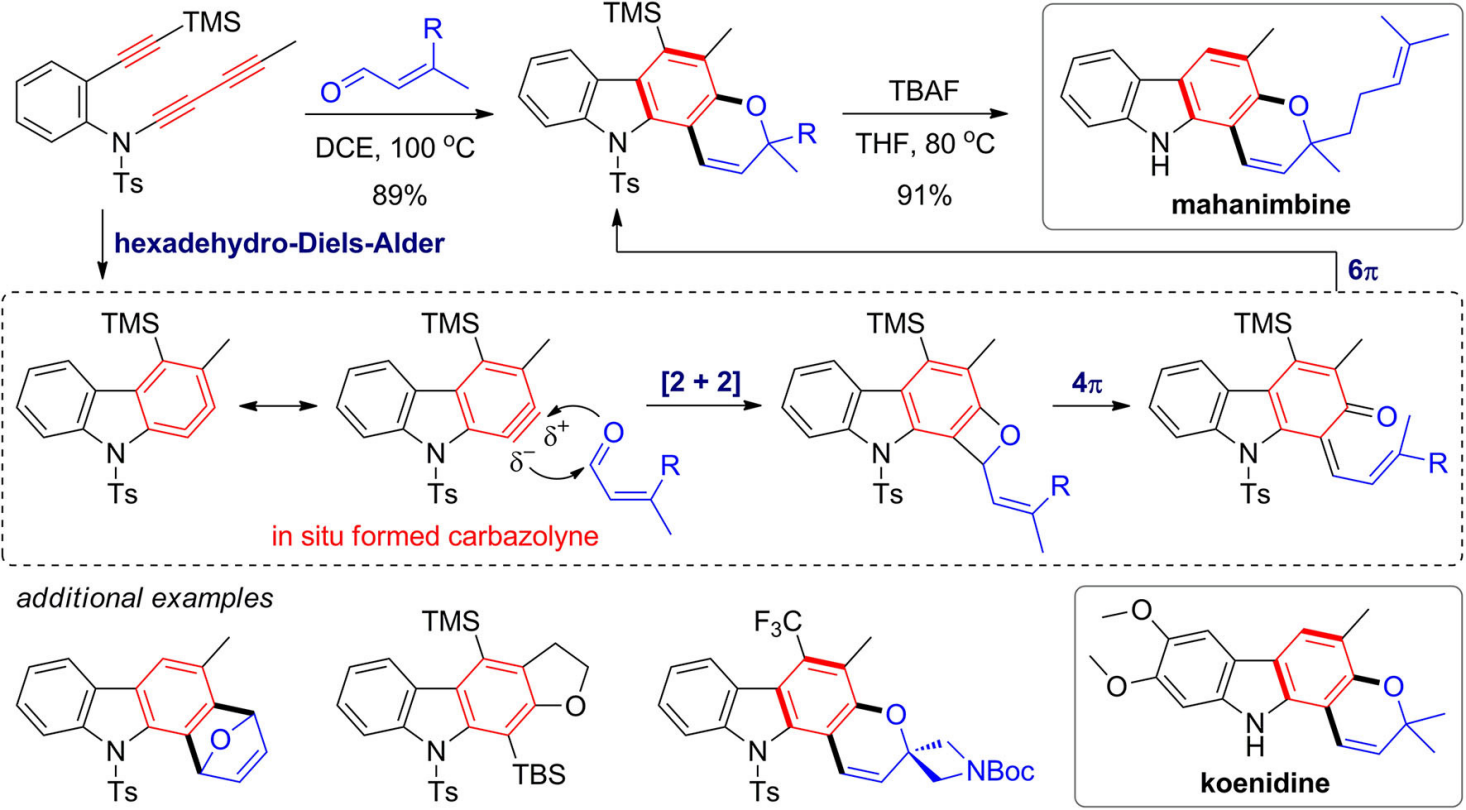
Synthesis of fused carbazoles via hexadehydro-Diels–Alder reaction.

Metal-catalyzed [2 + 2 + 2] cyclotrimerization of alkynes has been widely investigated and proved to be powerful strategy for construction of complex natural products (Goh et al., 2015; Heinz and Cramer, 2015). Hexadehydro-Diels–Alder (HDDA) reaction (Hoye et al., 2012) represents an alternative method that enables a metal-free approach for construction of fused and functionalized aromatics from alkynes. In 2016, Wang and Hoye (2016) developed a novel strategy for one-pot assembly of pyranocarbazole framework via the HDDA reaction of triyne substrates and subsequent trapping of the *in situ* formed carbazolyne by enals (Figure 8). The trapping reaction is proposed to proceed via a [2 + 2] reaction of a 1,3-zwitterion intermediate, followed by 4π and 6π electrocyclic reactions through benzoxetene and quinonemethide intermediates. By deprotection of both TMS group and Ts group in one pot, the obtained pyranocarbazoles can be converted to mahanimbine and koenidine, which are two curry tree alkaloids. Various fused carbazoles can be also obtained by different intramolecular or intermolecular trapping reactions.

**Figure 9:**
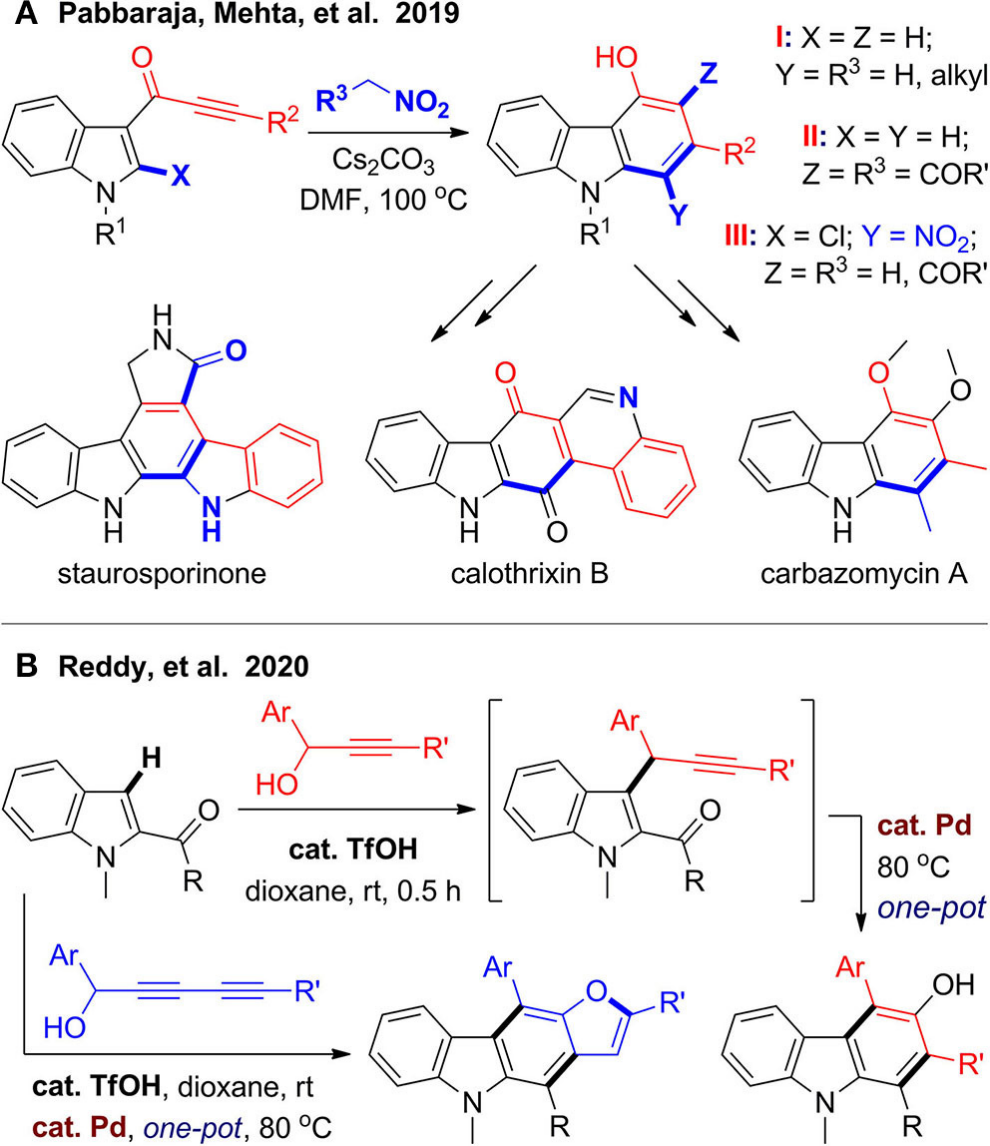
One-pot synthesis of hydroxycarbazoles.

In 2019, Singh et al. (2019) developed an efficient synthesis of diverse 4-hydroxycarbazoles via one-pot benzannulation from indole-3-ynones and nitromethane derivatives (Figure 9A). With indole-3-ynones as substrates and alkyl-substituted nitromethanes as a C1 synthon, a [5 + 1] cyclization proceeds under base-promoted condition to provide 1,2,4-trisubstituted carbazoles (type I), and one of them can be used for synthesis of carbazomycin A. While coupling with ethyl nitroacetate or benzoylnitromethane, an additional rearrangement step is involved, and 2,3,4-trisubstituted carbazoles are obtained (type II). When the reaction extends to 2-chloroindole-3-ynones under the same condition, the nitro group can be kept in the products (type III), which facilitates further derivation to access to polycyclic alkaloids such as calothrixin B and staurosporinone. Very recently, Reddy et al. (2020) developed a one-pot two-stage synthesis of 3-hydroxycarbazoles, which is also a ubiquitous structural motif in natural products (Figure 9B). In the first stage, coupling reaction between indole-2-carbonyls and 1-aryl propargylic alcohols was promoted by acid. After stirring at room temperature for 30 min, palladium catalyst was added to trigger hydroxylative benzannulation in one pot. Notably, when 2,4-diyn-1-ols were used as coupling partners, the coupling products underwent a cascade alkyne annulation to furnish furanocarbazole.

### Polycyclic Heterocycles With sp^3^ Centers

**Figure 10:**
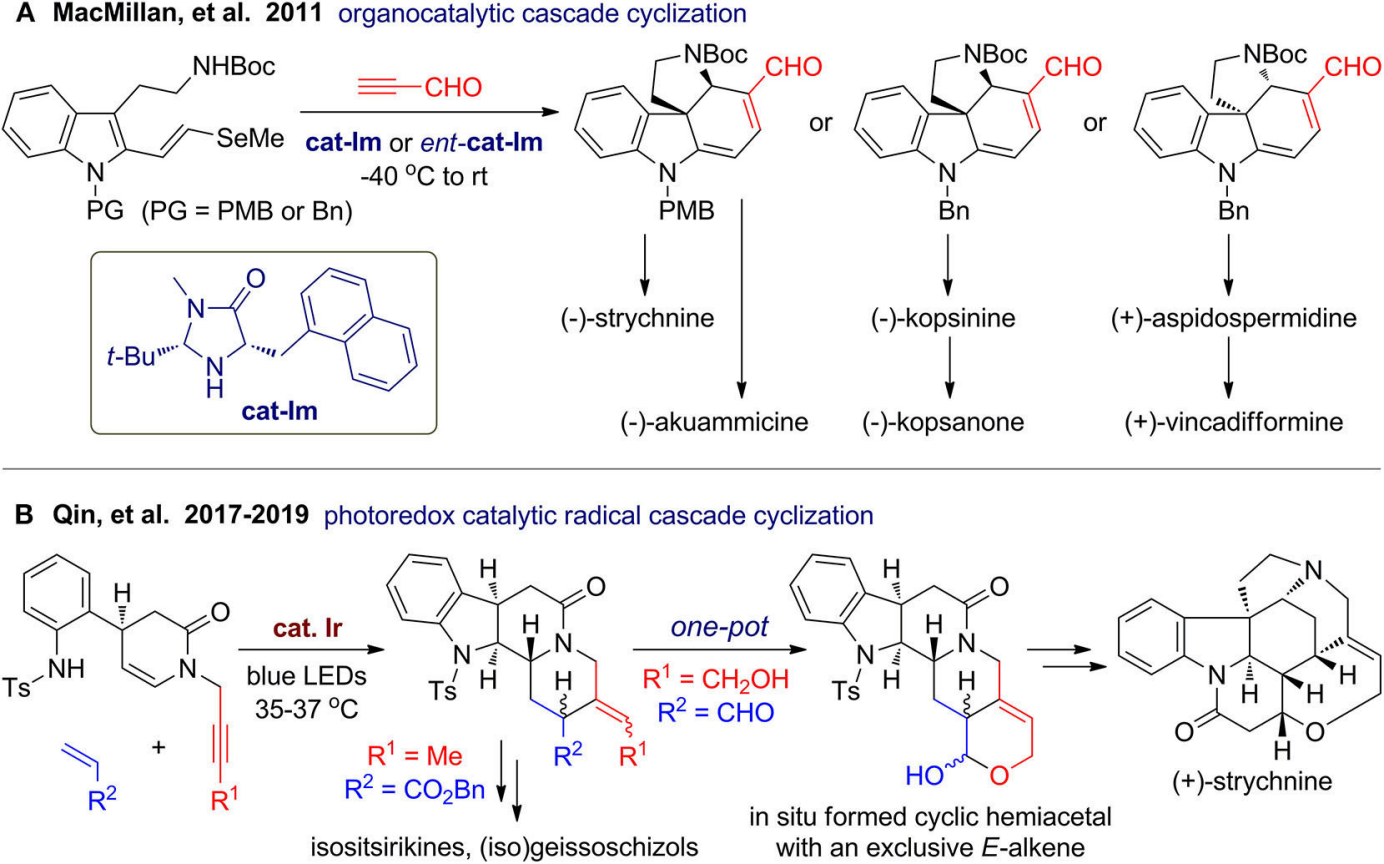
One-pot construction of polycyclic scaffolds for collective synthesis of alkaloids.

Collective total synthesis of a family or different classes of natural products from a common intermediate has emerged as a powerful strategy for improving product diversity along with synthetic efficiency (Mizoguchi et al., 2014; Li et al., 2018). This strategy is especially promising for polycyclic indolines, which are a big family of alkaloids with sp^3^ centers. When several classes of natural products share common biosynthetic intermediates, they usually have some highly resembling or related substructures and stereochemistry. In 2011, MacMillan's group (Jones et al., 2011) reported an impressive collective syntheses of natural polycyclic indolines including the well-known strychnine (Figure 10A). By coupling of *N*-protected and 2-functionalized tryptamines with propynal, a common tetracyclic intermediate can be obtained via organocatalytic asymmetric cascade reaction involving consecutive Diels–Alder cycloaddition, β-elimination, and amine conjugate addition. Starting from the common intermediates, total syntheses of six polycyclic indole alkaloids are finished in concise routes and improved overall yields compared to previous reports.

In 2017, Qin's group (Wang X. et al., 2017) developed a versatile iridium-catalyzed photoinduced radical cascade cyclization for rapid construction of polycyclic *N*-heterocycles, in which alkynes or alkenes were used as coupling partners or functional groups in multifunctional substrates. By using a rational designed multifunctional substrate, a tetracyclic scaffold can be constructed via intramolecular/intermolecular/intramolecular cascade (Figure 10B). Interestingly, although several *Z, E*-isomers and diastereomers were formed, they precisely satisfy the intrinsic stereochemical diversity of the target alkaloid family. In 2019, they (He L. et al., 2019) further extend this reaction for asymmetric total synthesis of (+)-strychnine, in which an alkynol-tethered substrate was employed to couple with acrolein. *In situ* formation of a cyclic hemiacetal may serve as a driving force for the *Z*-to-*E* isomerization to give the product with desired geometry. By the middle of 2019, they accomplished asymmetric total synthesis of more than 40 alkaloids belonging to seven families (Liu and Qin, 2019).

**Figure 11:**
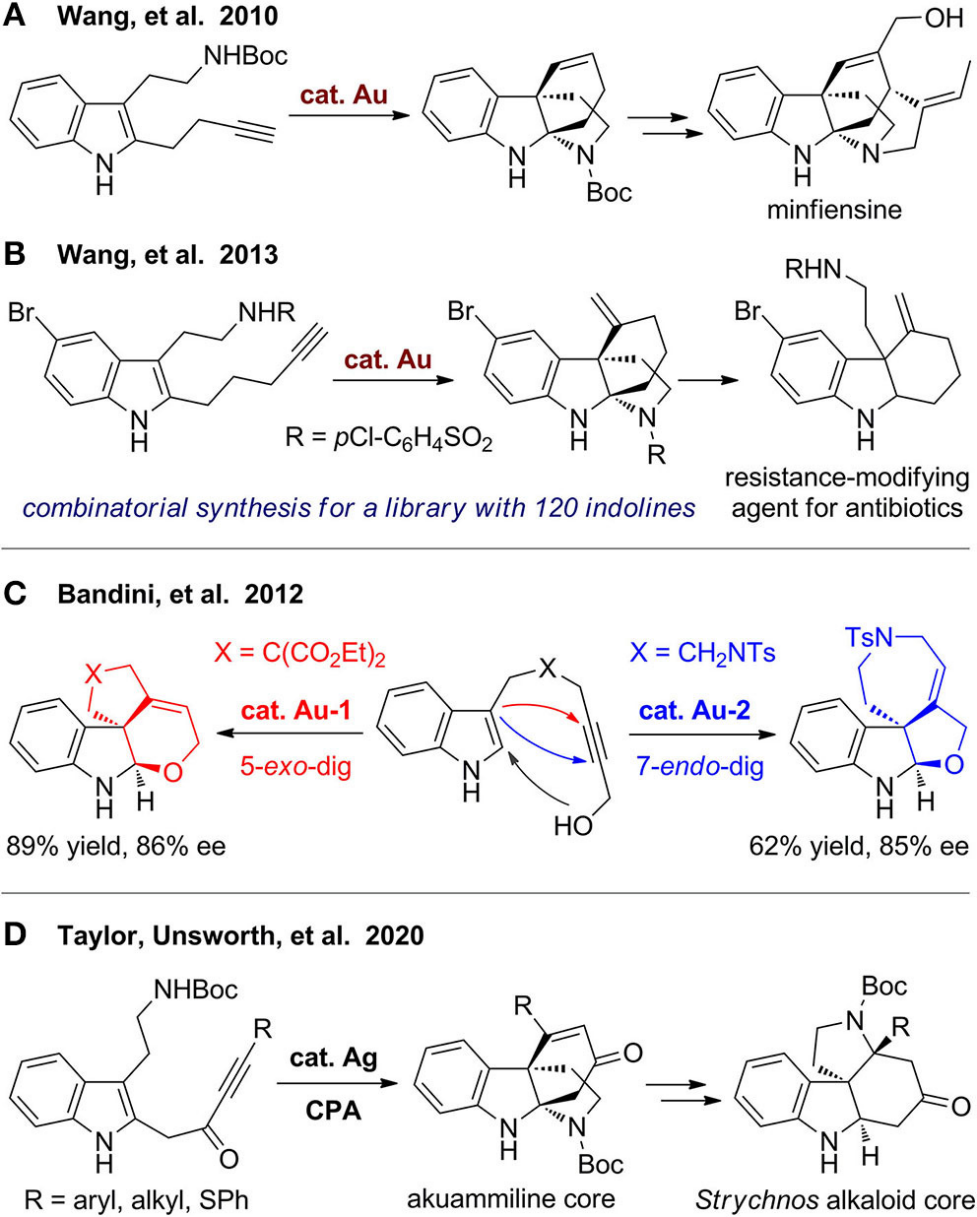
Syntheses of polycyclic indolines from alkyne-tethered indoles.

In 2010, Wang's group (Liu et al., 2010) developed gold(I)-catalyzed cascade cyclization of alkyne-tethered indoles to construct polycyclic indolines, which can be used for formal synthesis of minfiensine (Figure 11A). They (Podoll et al., 2013) further extended the methods by using a bioinspired assembly–cyclization–modification strategy to build a polycyclic indoline library including 120 diverse members with 26 distinct skeletons. In the assembly step, alkynyl indole substrates are obtained via a one-pot three-component coupling reaction. The reaction has poor selectivity, and two types of regioisomers were obtained. But on the other hand, the diversity increases due to the different reaction pathway, as the two types of products can be separated, and both are useful for the next step. These substrates were then subjected to gold(I)-catalyzed cascade cyclization to furnish polycyclic indolines, which underwent diverse derivations for structural modification and functionalization to obtain a library of natural product–like molecules. These polycyclic molecules were further employed for screening of resistance-modifying agent for antibiotics to fight against resistant bacteria. A tricyclic indoline was finally discovered, which have unique ability to selective enhance the activity of β-lactam antibiotics (Figure 11B).

Cera et al. (2012) reported a divergent and enantioselective gold(I)-catalyzed synthesis of highly functionalized polycyclic indolines via hydroindolination and *in situ* iminium trapping (Figure 11C). By using different gold catalysts and ligands, fused furoindolines and dihydropyranylindolines were obtained from substrates with different tethers via 5-*exo*-dig or 7-*endo*-dig cyclization, respectively. Recently, Rossi-Ashton et al. (2020) reported a silver(I)/chiral phosphoric acid–catalyzed cascade dearomative cyclization for modular assembly of the bridged tetracyclic core of the akuammiline alkaloids (Figure 11D). This scaffold can be further converted to fused *N*-heterocyclic framework, which is widely present in various *Strychnos* alkaloids.

**Figure 12:**
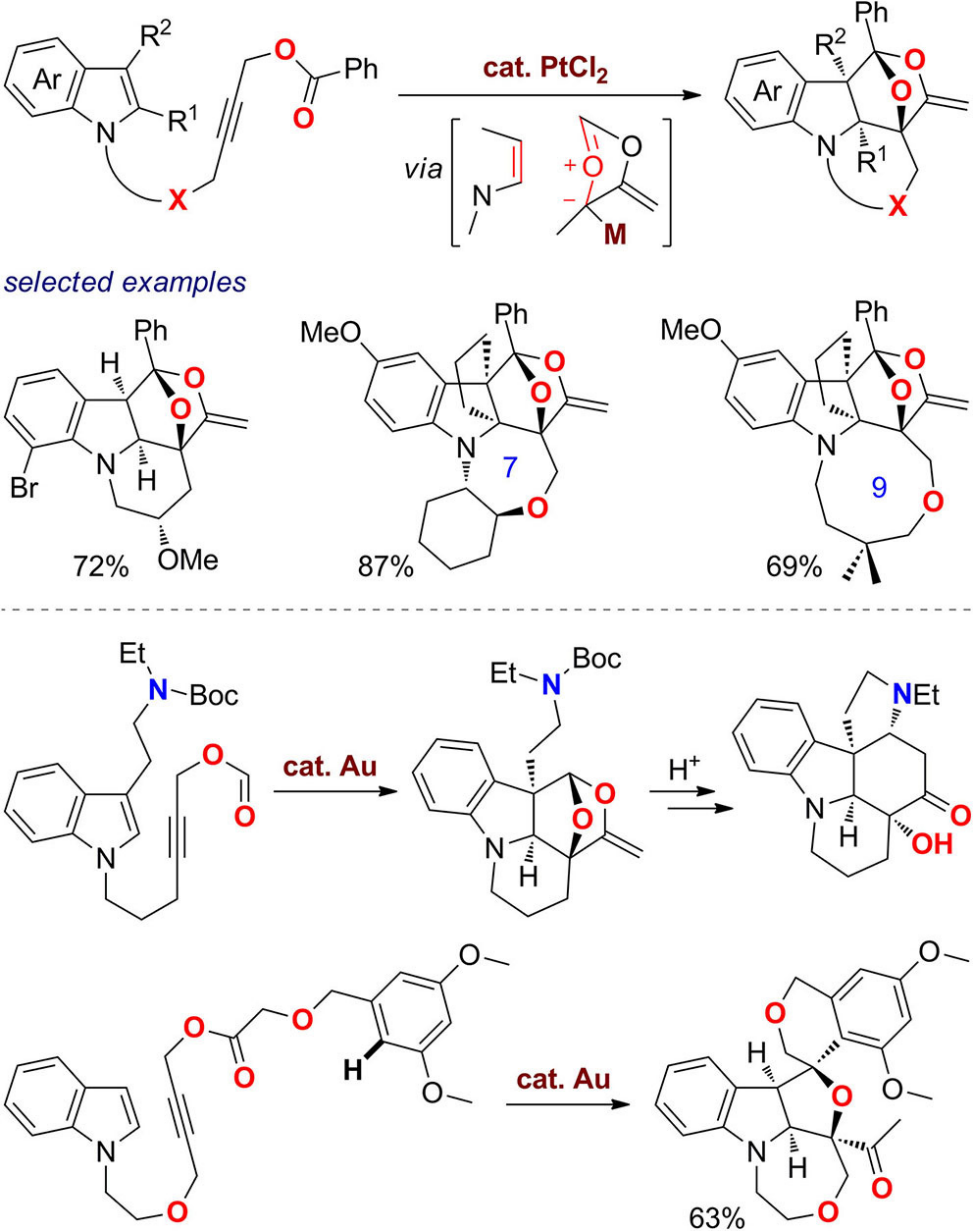
One-pot syntheses of oxa-bridged polycyclic indolines.

In 2018, Zheng's group (Wang et al., 2018) reported an efficient synthesis of oxygen-bridged indolines with diverse fused rings (Figure 12). With PtCl_2_ as catalyst, various indole *N*-tethered propargylic carboxylates undergo cascade reaction involving 1,2-acyloxy migration and intramolecular [3 + 2] cycloaddition to afford polycyclic indolines. Products with six- to eight-membered fused rings were obtained in good yields, and those with nine- to ten-membered rings can also be constructed successfully. With gold(I) as catalyst, a tryptamine-derived formate substrate undergoes the reaction smoothly, and the obtained oxa-bridged product can be further transformed toward the pentacyclic strychnine core via a two-step acid-promoted progress. Interestingly, a subsequent Friedel–Crafts reaction of the obtained enol ketal also occurred when the oxonium cation was trapped *in situ* by an aromatic nucleophile to furnish a complex fused and spiro polycyclic ring system.

Multifunctional indole substrates used in the above works usually need two to four steps for preparation. In 2012, Eycken and coworkers (Modha et al., 2012) employed Ugi four-component reaction for one-pot assembly of alkyne-functionalized indoles, which were subjected to gold(I)-catalyzed cascade cyclizations for rapid construction of tetracyclic indolines. Recently, they (He et al., 2018; He Y. et al., 2019) designed and synthesized two types of alkyne- and phenol-functionalized indoles by Ugi reaction. These multifunctional substrates were then used for construction of diverse polyheterocyclic scaffolds, including heteroarene-fused tricyclic heterocycles and indoline-fused caged molecules via gold(I)-catalyzed cascade reactions involving dearomative spirocarbocyclization. Although the obtained diverse polyheterocyclic scaffolds are not found in natural products so far, they can be treated as alkaloid mimics assembled by privileged substructures with the same level of architecturally complexity of PNPs.

**Figure 13:**
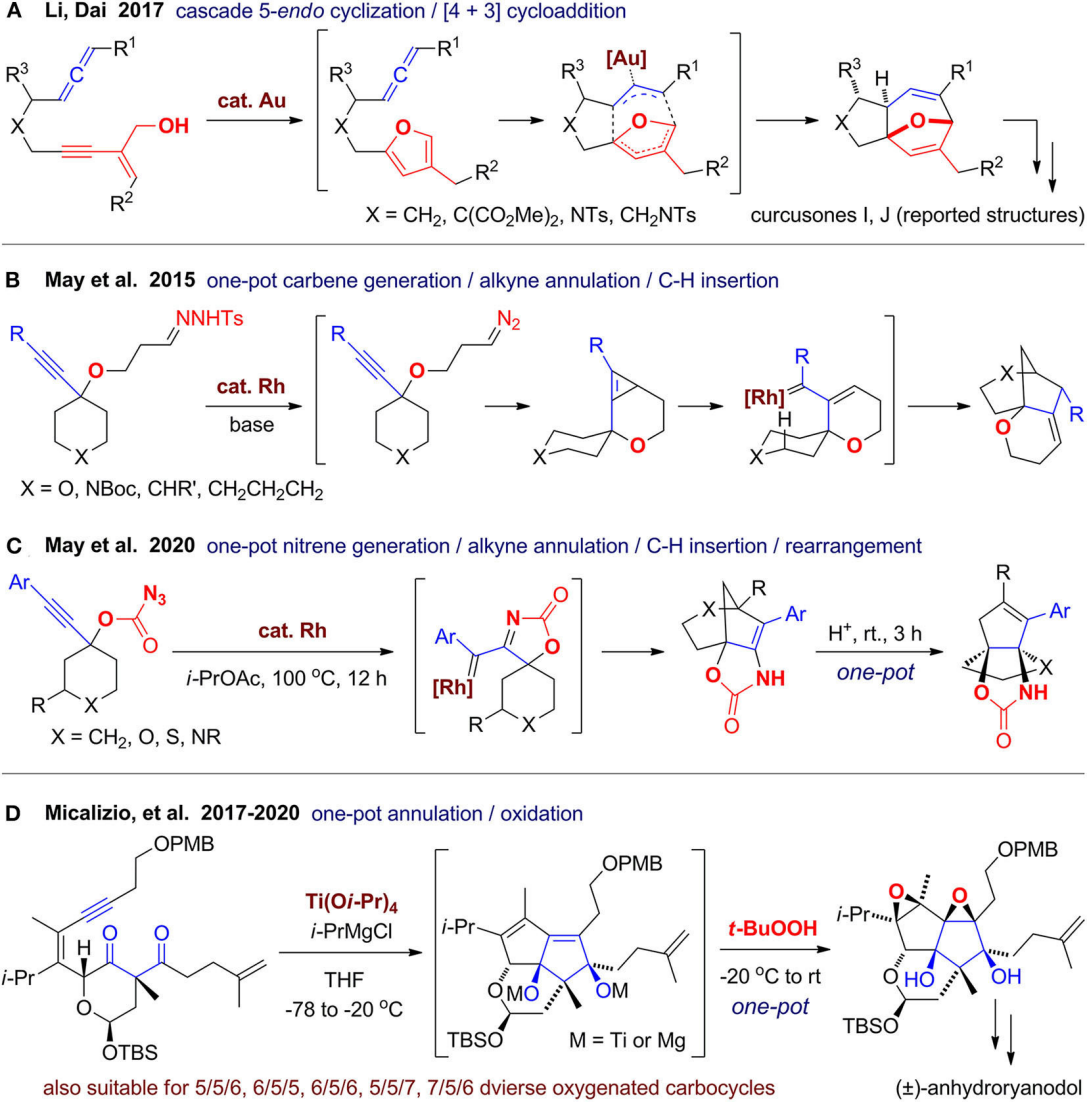
One-pot syntheses of oxygen-rich polycyclic scaffolds.

In 2017, Li and Dai (2017) reported a gold(I)-catalyzed cascade cyclization for rapid assembly of oxa-bridged tricyclic molecules, which was further used for synthesis of the reported curcusones I and J (Figure 13A). They have previously developed a stepwise approach for construction of kirkinine core via sequential furan formation and [4+3] cycloaddition (Li et al., 2017). In this work, they merged the two gold(I)-catalyzed steps in one-pot by using JohnPhosAuNTf_2_ as a single catalyst. When the furan ring is formed *in situ* via 5-*endo*-dig cyclization and isomerization of the enyne alcohol, it can be trapped by the allene group to construct the 5/7-fused ring system with an oxa-bridge. This reaction has a broad scope and can be scaled up to 5 g, which may facilitate its application for synthesis of related diterpenes and construction of natural product–like library.

In 2012, May's group (Jansone-Popova and May, 2012) developed a strategies for the construction of oxygen-containing bridged polycyclic ring systems via rhodium(II)-catalyzed carbene/alkyne cascades terminated by C–H insertion. In 2015, this strategy was successfully extended by employing tosyl hydrazones as carbene precursor (Le and May, 2015), which can be converted to diazoalkanes promoted by NaOSiMe_3_ (Figure 13B). The *in situ*–generated diazoalkanes then reacts with the catalyst to afford the rhodium carbene, which reacts with alkyne to give a fused cyclopropene intermediate. The cyclopropene ring then opened by rhodium followed by insertion of C(sp^3^)–H bond to furnish polycyclic product. By tuning the linker in the substrates, diverse bridged, fused, spiro, or even caged products with at least one oxygen atom in the rings can be obtained. Very recently, they (Wang and May, 2020) further developed a rhodium(II)-catalyzed nitrene/alkyne cascade reaction for synthesis of bridged heterocycles (Figure 13C). Although the yields are only moderate, the structural diversity is remarkable with least one oxygen atom and one nitrogen atom in the products. Furthermore, heteroatom-rich propellanes can be obtained via further rearrangement by direct addition of acetic acid or trifluoroacetic acid in one pot after the first step was finished.

In 2011, Dudley's group (Yang et al., 2011) reported a method for rapid construction of oxygen-rich caged core of aldingenin B via oxidative cycloketalization of alkyne-diol substrate by treatment with diphenyl diselenide and ammonium persulfate. Inspired by oxygen-rich PNPs such as ryanodol, Micalizio's group (Kier et al., 2017) developed a titanium(IV)-mediated coupling reaction of alkynes with 1,3- or 1,4-dicarbonyls. When the reaction is conducted using multifunctional substrates in an intramolecular fashion, fused carbocycles with two hydroxyl group could be obtained. When the reaction mixture is quenched by *t*-BuOOH in one pot, stereodefined epoxide product can be obtained (Du et al., 2018). Very recently, they (Du et al., 2020) accomplished the total synthesis of anhydroryanodol, which is also the formal total synthesis of ryanodol. The key step is the titanium(IV)-mediated intramolecular coupling of a well-designed diketone-enyne substrate with subsequent oxidative quenching in one-pot (Figure 13D). Although the unoptimized yield was only 23% to 33%, polycyclic scaffold with well-arranged functional groups and six contiguous stereocenters can be constructed in one shot. They (Karmakar et al., 2019) also investigated this strategy using various substrates to forge diverse polycyclic ring systems, including those with a seven-membered ring, which demonstrate a convincing example that new synthetic methods developed targeting specific natural products can be also used for generating molecular diversity.

## Summary and Perspectives

In the past 10 years, significant progress has been made for construction of PNP scaffolds with high step and atom economies, as well as structural complexity and diversity. Among a range of different synthetic methods, several types of alkyne-anticipated one-pot annulative reactions are emerged and well-tested to be efficient and versatile for rapid assembly of diverse skeleton. These strategies may be treated as candidates of “privileged synthetic strategies” for “privileged polycyclic scaffolds.” For construction of polycyclic carbocycles (sometimes with tethered or bridged heteroatoms), alkynes are usually used together with alkenes, among which at least three types of synthetic methods can be tagged as privileged including gold-catalyzed ene–yne cycloisomerizations, domino RCM, and cascade metal-catalyzed cyclization/pericyclic reaction. Various alkyne-anticipated synthetic methods have been developed for rapid construction of diverse polycyclic heterocycles, among which at least two strategies have proved to be versatile. One is the combination of C–H functionalization and alkyne annulation of multifunctional substrates, including construction of fused pyridines and pyrroles via cascade C–H activation/alkyne annulation triggered by a nitrogen-containing group such as amino, oxime, or azide, as well as construction of bridged polyheterocycles via cascade alkyne annulation/C–H insertion triggered by a hydrazone or an azide group. The other strategy is construction of polycyclic indolines via cascade dearomative cyclization of alkyne-tethered indoles.

Although the above one-pot scaffold methods are quite efficient, synthetic routes to the multifunctional substrates and post-modifications are not concise in many cases, thus lowering the step economy from raw materials to desired molecules. Therefore, the overall synthetic efficiency and economy should be taken into account besides those of the key step. The robustness of the reactions, especially the tolerance of air and water should also be taken into account, which will increase operational convenience especially for one-pot multistage reactions (Collins and Glorius, 2013; Zheng and Hua, 2014b; Chen et al., 2019). Also, more concern should be paid for the overall sustainability of the synthetic sequences including the renewability of the starting materials (Kühlborn et al., 2020). Furthermore, one-pot reactions, along with the preparation of substrates and post-functionalization, can be combined with flow chemistry and integrated into automatic syntheses (Jürjens et al., 2015; Coley et al., 2019; Bloemendal et al., 2020; Chatterjee et al., 2020; Nam et al., 2020), which should have new opportunities to further improvement of synthetic efficiency.

The power of nature for generation of diversity and complexity from simple building blocks under mild conditions is embodied by natural products, which will continuously stimulate chemists to develop new reactions. In the above works, a trend is observed that two or more synthetic concepts and strategies including target-, diversity-, function-, and biology-oriented synthesis are usually combined in construction of complex cyclic molecules. Natural product–like and drug–like molecules can further expand the degree of scaffold diversity beyond PNPs by adding or changing heteroatoms, tuning ring size and linkers, as well as assembly of privileged substructures. Empirical scaffold reproducing and mimicking of natural products may be upgraded to systematic scaffold analysis and rational design under the guidance of computational chemistry, big data, and artificial intelligence (Yang et al., 2019; Neto et al., 2020). With further development of the above methods and emergence of novel strategies and technologies, the application of alkyne-involved one-pot cyclizations will be expanded for more diverse polycyclic scaffolds and finally proved to be powerful synthetic toolkit for both natural products and medicinal chemistry.

## Author Contributions

LZ and RH proposed the theme and outline of this review. LZ collected and organized articles in literature with feedback from RH. LZ wrote the manuscript with revision by RH. All authors contributed to the article and approved the submitted version.

## Conflict of Interest

The authors declare that the research was conducted in the absence of any commercial or financial relationships that could be construed as a potential conflict of interest.
